# Lower LEDD and Polypharmacy Rates Beyond the Honeymoon Period in Patients With Parkinson's Disease Integrative Western–Korean Medicine Interventions: A CARE-Compliant Case Series

**DOI:** 10.1155/padi/9860808

**Published:** 2025-10-28

**Authors:** Cheol-Hyun Kim, Taeseok Ahn, Youngjo So, Hyeon-Gyu Cho, Jiwoo Kim, Jihyun Moon, Myungjin Oh, Sunny Kang, Sangho Ji, Linae Kim, Sangkwan Lee, Namkwen Kim

**Affiliations:** ^1^Department of Internal Medicine, College of Korean Medicine, Wonkwang University, Iksan 54538, Republic of Korea; ^2^VARO Korean Medicine Clinic, Seoul 07714, Republic of Korea; ^3^Department of Acupuncture & Moxibustion Medicine, College of Korean Medicine, Pusan National University, Yangsan 50612, Republic of Korea; ^4^Keumkang Korean Medicine Clinic, Cheongju 28145, Republic of Korea; ^5^Research Institute for Korean Medicine, Pusan National University, Yangsan 50612, Republic of Korea; ^6^Center for Big Data and Comparative Effectiveness Research, Economic Evaluation in Health and Medicine, Institute of Korean Medicine, Pusan National University, Yangsan 50612, Republic of Korea

**Keywords:** integrative treatment, levodopa equivalent daily dose, Parkinson's disease, polypharmacy

## Abstract

**Background:**

Parkinson's disease (PD) is the second most common neurodegenerative disorder with significant social costs, mainly owing to hospitalization, which is frequently associated with high levodopa equivalent daily dose (LEDD) and polypharmacy rather than neurological symptoms alone. Integrative treatment combining Western and Korean medicine may help control these factors and reduce the need for hospitalization. We investigated the potential impact of integrative treatment on LEDD and polypharmacy in patients with PD > 5 years postdiagnosis.

**Methods:**

Fifteen patients with PD (KCD code G20), diagnosed > 5 years earlier, who received integrative treatment at Gwangju Korean Medicine Hospital, Wonkwang University, from April 1, 2022, to July 30, 2024, were enrolled. A retrospective chart review was conducted to collect demographic and clinical data, including LEDD, medication count, and treatment details. Summary statistics were presented as median (IQR) and mean ± SD.

**Results:**

In the integrative treatment cohort, the prevalence of both LEDD and polypharmacy was lower than that in studies involving conventional treatment alone. The mean LEDD was 321.71 (median, 200.0) mg, while only two patients exceeded the LEDD threshold of 300 mg, which was associated with motor complications. Polypharmacy was observed in 13.3% of patients and hyperpolypharmacy in 6.7%, representing lower proportions compared with previous reports on conventional treatments. Representative cases highlighted symptom improvement and a reduced need for medication with integrative approaches, particularly acupuncture and herbal medicine.

**Conclusion:**

These findings suggest that integrative treatment may contribute to lowering LEDD and medication counts in patients with PD, which could potentially reduce hospitalization rates and the associated social costs. Further prospective studies comparing the integrative and nonintegrative treatment groups are needed to clarify these findings and evaluate the role of integrative treatment in the long-term management of PD.

## 1. Introduction

Parkinson's disease (PD) is the second most common neurodegenerative disorder and is ranked after Alzheimer's disease. Despite treatments for symptom alleviation, PD has no cure and it confers substantial social costs [1]. According to data from the Health Insurance Review and Assessment Service (HIRA) in Korea, hospitalization costs comprise 85.5% of the total care cost for patients with PD [2]. Reducing the need for hospitalization could potentially decrease the associated social costs [3]. Approximately 15% of hospitalizations of patients with PD are attributable to the neurological symptoms of the disease itself, and the majority of hospitalizations are related to complications or medication-related side effects [3]. The levodopa equivalent daily dose (LEDD) and polypharmacy (or hyperpolypharmacy) are the two major factors that are strongly associated with the hospitalization frequency of patients with PD [4, 5]. The LEDD is a standardized measure that converts the total dose of dopaminergic medication into an equivalent levodopa dose to facilitate the standardized comparison of treatment effects. For example, 1 mg pramipexole is equivalent to 100 mg levodopa [6]. Increased LEDD correlated with a significant increase in dyskinesia, wearing-off phenomena, and other motor complications, all of which are closely linked to hospitalization in patients with PD [7].

Polypharmacy and hyperpolypharmacy are defined as the daily use of five or more and ten or more medications, respectively. A systematic review and meta-analysis conducted in 2022 found that 40% and 18% of patients with PD aged ≥ 65 years experienced polypharmacy and hyperpolypharmacy, respectively [5]. Furthermore, up to 90% of patients with PD aged ≥ 65 years may experience polypharmacy [8]. The meta-analysis found a strong correlation between hospitalization and both polypharmacy and hyperpolypharmacy in patients with PD, and the disease-related risk doubled with the increase in the number of medications [5].

The 2023 HIRA data in Korea show that 81.1% of patients with PD who received conventional medical treatment alone required hospitalization, whereas 90% of patients who received Korean medicine were managed on an outpatient basis [2]. As most patients in the Korean medicine group also take dopaminergic medications, they receive integrative treatment that combines Western and Korean medicine. These HIRA data indicate that integrative treatment may be associated with a lower frequency of hospitalization, which potentially reduces the hospitalization-related social costs [2].

Patients with PD typically experience a “honeymoon period” of optimal medication efficacy that lasts for up to 5 years; thereafter, the medication requirements frequently increase [9]. However, our preliminary observations of patients who received integrative treatment for more than 5 years suggested a different trend, with these patients exhibiting lower LEDD and polypharmacy rates than those of similar patients with chronic PD [10]. The current literature [11, 12] indicates that integrative treatment can help maintain consistent levodopa blood levels or significantly improve UPDRS scores. However, to our knowledge, no prior study has reported an association between integrative treatment and reduced LEDD and polypharmacy.

To address this knowledge gap, this case series retrospectively examined patients with PD who had received integrative treatment for more than 5 years. Through detailed case descriptions and analyses, we aimed to explore the potential association between integrative treatment and the prevention of increases in LEDD and medication burden.

## 2. Methods

### 2.1. Study Design and Setting

This retrospective case series was conducted at Gwangju Korean Medicine Hospital, Wonkwang University, Republic of Korea. The study period covered patient visits from April 1, 2022, to July 30, 2024, which corresponds to the duration of the 4^th^ phase of the Western and Korean Medicine Collaborative Pilot Program.

### 2.2. Study Population and Selection Criteria

#### 2.2.1. Inclusion Criteria

Patients were included if they met all of the following criteria:

• The diagnosis of PD was confirmed by a neurologist according to the UK Brain Bank criteria with the Korean Classification of Diseases (KCD) code G20.
• They had a disease duration of more than 5 years from the initial diagnosis
• They received integrative treatment at our institution during the study period
• Their complete medical records, including medication history and treatment details, were available

#### 2.2.2. Exclusion Criteria

Patients were excluded if they had:

• Secondary parkinsonism or atypical parkinsonian syndromes
• Incomplete medical records or missing essential data (LEDD calculation, medication count)

#### 2.2.3. Definition of Integrative Treatment

For this study, integrative treatment was defined as the concurrent use of:

• Conventional Western medical treatment for PD (including dopaminergic medications prescribed by neurologists)
• Korean medicine interventions administered at our institution, including:
  - Acupuncture therapy (i.e., manual acupuncture, electroacupuncture, or pharmacopuncture)
  - Korean herbal medicine (individualized herbal formulations)
  - Other Korean medicine modalities (cupping, moxibustion, etc.)
• Care coordinated between Western medicine physicians and Korean medicine practitioners

### 2.3. Data Collection and Outcome Measurements

Through a comprehensive retrospective chart review, the following data were systematically collected.

#### 2.3.1. Clinicodemographic Characteristics

• Age (in years) at the initial visit and sex.
• Disease duration (time from initial PD diagnosis)
• Duration of integrative treatment (time from first Korean medicine consultation)
• Comorbidities, classified by organ system

#### 2.3.2. Primary Outcome Measures

• LEDD: This was calculated using established conversion factors for all dopaminergic medications, including levodopa/carbidopa, dopamine agents (i.e., pramipexole, ropinirole, and rotigotine), monoamine oxidase-B inhibitors (i.e., selegiline and rasagiline), catechol-O-methyltransferase inhibitors (i.e., entacapone), and amantadine.
• Medication count: The total number of prescribed medications (both Western and Korean) that were being taken daily at the last visit.
• Polypharmacy: Defined as the concurrent use of more than five medications daily
• Hyperpolypharmacy: Defined as the concurrent use of more than ten medications daily

#### 2.3.3. Secondary Outcome Measures

• Hoehn and Yahr (H&Y) stage at the initial and last visits
• Details of Korean medicine treatments that were received
• Adverse events related to integrative treatment

#### 2.3.4. Data Collection Process

All data were collected at the most recent visit during the study period. Two independent reviewers extracted data using a standardized form, and discrepancies were resolved through discussions with a senior investigator.

### 2.4. Statistical Analysis

Descriptive statistics were used to summarize the patient characteristics and outcomes. Given the small sample size and potential non-normal distribution of continuous variables, both parametric and nonparametric measures were reported. Continuous variables are presented as both mean ± standard deviation and median with interquartile range (IQR). Categorical variables are presented as frequencies and percentages. No imputation was performed for missing data, and the number of available observations was reported for each variable.

All statistical analyses were performed using the R software (Version 4.3.2; R Foundation for Statistical Computing, Vienna, Austria).

Considering the descriptive nature of this case series, no inferential statistical tests were performed. This study described clear patterns to generate hypotheses for future comparative studies.

## 3. Results

### 3.1. Case Presentation Summary

Seven patients met the inclusion criteria, and their clinicodemographic characteristics are shown in Table 1. Two patients were younger than 65 years and five were aged 65 years or older; three patients were male, and four were female. The duration of PD was between 5 and 10 years in three patients and more than 10 years in four patients. All seven patients began integrative treatment within 5 years of PD diagnosis. The H&Y stages were as follows: Stage 1, one patient; Stage 2, one patient; Stage 3, four patients; and Stage 4, one patient. Regarding comorbidities, metabolic disorders were the most common and affected four patients, followed by cardiovascular disorders in three patients, psychiatric disorders in two patients, and neurological and urological disorders in one patient each.

All patients were maintained on stable dopaminergic medication regimens at baseline, with the individual LEDD values documented in Table 2. Electroacupuncture is universally administered as a primary Korean medical intervention targeting parkinsonian motor symptoms. Additional Korean medical therapies were individualized to address accompanying nonmotor symptoms and complications that commonly contribute to polypharmacy in patients with PD. These include bee venom pharmacopuncture for enhanced motor function support and gastrointestinal-targeted herbal medicines that were specifically selected to optimize levodopa absorption and bioavailability. The strategic focus on nonmotor symptoms was based on evidence that the majority of the polypharmacy burden in PD stems not only from dopaminergic medications but also from medications prescribed to manage associated symptoms, such as pain, gastrointestinal dysfunction, and other comorbidities. By addressing these nonmotor symptoms through Korean medicine-based interventions, the treatment approach aims to maintain or improve motor control while preventing LEDD escalation and reducing the overall medication burden. This strategy addresses both primary parkinsonian symptoms and the broader medication complexity that is typical of long-term PD management.

### 3.2. Representative Cases

#### 3.2.1. Case 1: Acupuncture-Mediated Headache Control Leading to Polypharmacy Reduction

**Figure 1:**
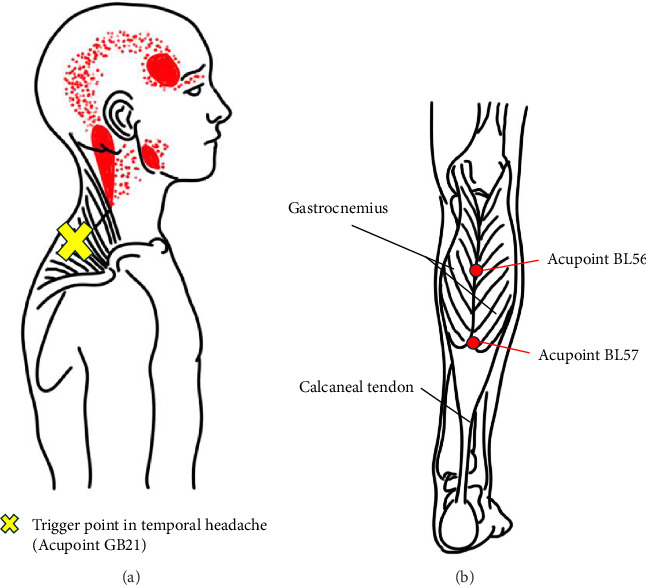
Acupoints utilized for targeted pain management to reduce polypharmacy. (a) GB 21 (Jianjing) acupoint for temporal headache control in Case 1. Electroacupuncture (EA) at this trigger point led to the discontinuation of gabapentin, tramadol, and acetaminophen by effectively managing headaches that were refractory to conventional analgesics and botulinum toxin injections. (b) BL56 (Chengjin) and BL 57 (Chengshan) acupoints for nocturnal leg pain control in Case 2. EA at these bladder meridian points enabled discontinuation of pregabalin, tramadol, and piroxicam by successfully managing severe nocturnal leg pain (NRS 9 ⟶ 2) that was refractory to complex polypharmacy regimens. Both interventions demonstrate how the precise selection of acupoints can replace multiple analgesic medications with nonpharmacological alternatives, and thereby directly contribute to polypharmacy reduction in patients with Parkinson's disease.

A 64-year-old man with a 7-year history of PD presented with three major symptoms (resting tremor, bradykinesia, and rigidity), along with severe intermittent headaches in the temporal region. The patient's polypharmacy burden was significantly increased by these severe headaches, which necessitated gabapentin 100 mg once daily and tramadol hydrochloride 37.5 mg, and acetaminophen 325 mg PRN, along with three botulinum toxin injection sessions that were administered at 3-month intervals; the headaches were refractory to all of these treatments. We assessed the headache as a referred pain owing to muscle tension and identified trigger points in the trapezius muscles. Acupuncture was administered at the trigger point (acupoint GB21, Jianjing) using a 0.25 × 30 mm needle (Dongbang Medical Co., Ltd.; Figure 1(a)). During needle insertion, the patient was instructed to achieve a *de-qi* sensation, followed by 2–3 manipulations after *de-qi* was achieved. Without removing the needle, electroacupuncture stimulation (4 Hz, tolerable electrical current strength) was applied for 20 min. The treatment was administered twice a week for 2 weeks. After four acupuncture sessions, the headache intensity decreased from 8 to 3, and the frequency decreased from once daily to approximately once every 4–5 days. Importantly, this symptom improvement enabled complete discontinuation of both the gabapentin and tramadol–acetaminophen combination without any adverse effects. This acupuncture intervention directly reduced the patient's medication burden by eliminating two analgesic medications while providing superior headache control.

#### 3.2.2. Case 2: Acupuncture-Mediated Nocturnal Leg Pain Control Leading to Polypharmacy Reduction

A 74-year-old man with a 12-year history of PD and typical motor symptoms developed severe nocturnal leg pain (NRS 9) that resulted from intense lower limb rigidity. This symptom required a complex pain-management regimen comprising 75 mg pregabalin twice daily, 50 mg tramadol hydrochloride twice daily, and intramuscular piroxicam (10 mg/mL) injections, as needed, during severe episodes. Despite this multidrug approach, no improvement was observed in the pain intensity, frequency, or lower limb rigidity. Acupuncture was administered in the prone position at BL56 (Chengjin) and BL57 (Chengshan) along the bladder meridian on the lower extremities (Figure 1(b)) using a 0.25 × 30 mm needle (Dongbang Medical Co., Ltd.). The treatments were performed twice daily at a depth of 2.5 cm, with electroacupuncture stimulation (4 Hz at a tolerable electrical current strength) applied for 20 min per session. After 1 week of continuous treatment, the nocturnal pain intensity decreased dramatically to NRS 2, which immediately eliminated the need for piroxicam injections. With ongoing acupuncture treatment, the patient was able to discontinue pregabalin and tramadol hydrochloride without any worsening of pain intensity or frequency. This acupuncture intervention achieved superior pain control while reducing the patient's medication count of all three drugs.

#### 3.2.3. Case 4: Integrative Treatment for LEDD Stabilization and Long-Term Progression Prevention

A 71-year-old woman with an 11-year history of PD presented with three major symptoms along with progressive bradykinesia. Her neurologist recommended an increase in the dopaminergic medication dosage to address symptom progression. However, the patient sought integrative treatment to avoid LEDD escalation. At baseline, the patient maintained a stable LEDD of 200 mg without recent dose adjustments. To stabilize plasma levodopa concentrations and provide potential neuroprotective effects, a combined integrative regimen was initiated, which included electroacupuncture using 0.25 × 30 mm needles (Dongbang Medical Co., Ltd.) with manual stimulation for 20 min, bee venom pharmacopuncture at GB34 (Yanglingquan), and *Bojungikgi-tang* (*Buzhong Yi Qi Tang,* in Chinese, and *Hochuekkito*, in Japanese) administered orally three times daily. Electroacupuncture and bee venom pharmacopuncture were performed twice a week for 8 weeks, and herbal medicine was continued throughout the study period. After 8 weeks of integrative treatment, the patient maintained her daily activities at the same baseline level without requiring an increase in LEDD. Remarkably, she continued this treatment regimen for seven consecutive years without clinical deterioration or escalation of dopaminergic medication.

#### 3.2.4. Case 5: Prevention of Inappropriate LEDD Escalation and Polypharmacy Reduction Through Drug-Induced Parkinsonism Management

A 67-year-old woman with a 9-year history of PD presented with well-controlled baseline symptoms. The patient experienced sudden worsening of perioral and left-hand tremors (NRS 2–5) after taking levosulpiride 25 mg three times daily for gastrointestinal symptoms, which could have led to inappropriate increases in dopaminergic medication. Upon discontinuation of levosulpiride, acupuncture at the four gate acupoints (LI4 and LR3) was administered daily for 20 min. After 3 days, the tremors returned to baseline (NRS 2), and the gastrointestinal symptoms improved.

#### 3.2.5. Case 6: Polypharmacy Reduction and LEDD Optimization Through Gastrointestinal Function Enhancement

**Figure 2:**
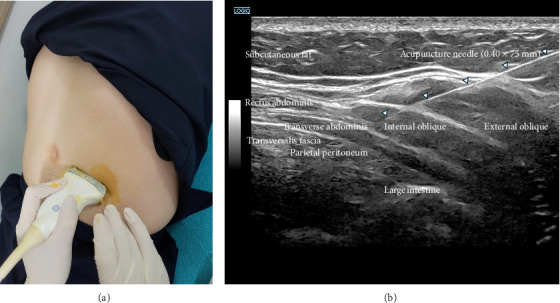
Ultrasound-guided acupuncture at SP14 for polypharmacy reduction and LEDD optimization through gastrointestinal function enhancement in Case 6. (a) Ultrasound-guided procedure at SP14 (Fujie). A GE LOGIQ FORTIS with an ML 6–15 MHz linear probe was used via a lateral approach with an in-plane view using a short-axis scan. Pre-procedurally, the probe was wrapped with a sterile drape and disinfected with povidone-iodine. The procedure site (SP14) was double disinfected with alcohol and povidone-iodine. A 0.40 × 75 mm needle was used for the procedure. (b) Ultrasound image of left SP14 during the ultrasound-guided procedure. To minimize the risk of infection, the needle was inserted only up to the vicinity of the transversalis fascia while ensuring that it did not come into direct contact with the large intestine. Subsequently, diaphragmatic breathing was encouraged to induce a *de-qi* sensation. This intervention achieved dual therapeutic benefits by enhancing gastrointestinal motility: complete discontinuation of four ineffective constipation medications (lactulose concentrate, magnesium hydroxide, and bisacodyl) following the first significant bowel movement in years within 6 h, and LEDD reduction from 300 to 150 mg through improved levodopa absorption and bioavailability while maintaining motor function. This case demonstrates how targeted gastrointestinal intervention can simultaneously reduce medication burden and optimize the efficacy of dopaminergic therapy.

A 64-year-old man with a 10-year history of PD presented with severe refractory constipation. The patient's medication burden was significantly increased by severe constipation requiring multiple ineffective medications, including lactulose concentrate (20.1 g), magnesium hydroxide (500 mg) thrice daily, and bisacodyl (10 mg) suppositories, as needed. Despite this multidrug regimen, the patient experienced persistent severe constipation with no bowel movement, and even finger enema attempts were unsuccessful. To directly stimulate intestinal function, ultrasound-guided manual acupuncture was performed at SP14 using a 0.40 × 75 mm needle (Dongbang Medical Co., Ltd.; Figures 2(a) and 2(b)). A GE LOGIQ FORTIS with an ML 6–15 MHz linear probe was used through a lateral approach with an in-plane view using a short-axis scan. The patient was instructed to practice diaphragmatic breathing for a 20-min duration of acupuncture to enhance intestinal motility stimulation. Six hours after a single ultrasound-guided acupuncture treatment, the patient achieved the first significant bowel movement. Subsequently, all previous constipation medications were completely discontinued and replaced with a single herbal medicine (*Gyeji-ga-jakyak-daehwang-tang*), which reduced the medication count from four constipation-related drugs to one and established regular bowel movements. Beyond polypharmacy reduction, this gastrointestinal function enhancement critically improved levodopa absorption and bioavailability, which enabled LEDD reduction from 300 to 150 mg through dosage reductions of pramipexole 0.25 mg, rasagiline 0.75 mg, and levodopa 50 mg while maintaining motor function.

### 3.3. Grouped Findings

Table 2 presents individual data for each patient, including LEDD and medication count.  Table 3 provides summary statistics of H&Y stage, LEDD, medication count, and duration of collaborative treatment with Western–Korean medicine (DCTWK). To accurately reflect the characteristics of the data, summary statistics were presented as both median (IQR) and mean ± SD, owing to the small sample size to ensure comprehensive data representation. At baseline visit, LEDD was median (IQR) 300.0 (250.0–477.5) mg and mean ± SD 386.43 ± 216.40 mg, which decreased to median (IQR) 200.0 (200.0–426.0) mg and mean ± SD 321.71 ± 219.21 mg at last follow-up visit. Moreover, the medication count decreased from baseline median (IQR) 7.0 (4.5–10.0) and mean ± SD 8.0 ± 4.93 to median (IQR) 4.0 (3.5–7.5) and mean ± SD 6.14 ± 4.06 at follow-up. The mean changes were as follows: LEDD reduction of 64.71 ± 62.97 mg, medication count reduction of 1.86 ± 1.35, and H&Y stage improvement of 0.14 ± 0.38. At baseline, four patients (57.1%) exceeded the LEDD threshold of 300 mg that is associated with motor complications, which decreased to two patients (28.6%) at follow-up. Polypharmacy affected six patients (85.7%) at baseline but only two patients (28.6%) at follow-up, with hyperpolypharmacy decreasing from two patients (28.6%) to one patient (14.3%).

The types of herbal medicines contributing to medication optimization included *Gyeji-ga-jakyak-daehwang-tang*, administered to four patients, and *Bojungikgi-tang*, administered to three patients; both medications were used to improve gastrointestinal function to alleviate dyspepsia or constipation, and thereby enhance levodopa absorption, maintain stable blood dopamine levels, ultimately reduce LEDD requirements, and decrease the dependence on multiple nondopaminergic medications.

### 3.4. Patients' Perspective

#### 3.4.1. Case 1

“I had an experience in which receiving treatment from both Western and Korean medicines helped me avoid potentially serious situations. One morning, I wake up and suddenly cannot move my bodies. Alarmed, I visited the emergency room of a university hospital. They performed brain MRI and blood tests, but since there were no significant abnormalities, they told me to go home and return to see my neurologist for an outpatient follow-up. Changing appointment dates at university hospitals is difficult. Since I was not receiving any specific treatment from the hospital, I decided to come here, where I had been receiving Korean medical treatment for a long time. When I explained my symptoms, the first thing I was asked about how I was taking my medication. Because I had Parkinson's disease, I took levodopa but stopped taking it a few days prior because of severe constipation. I was not even sure that it was working, so I stopped taking it. This is why I cannot move. However, Western medicine doctors, who rely solely on machines, have missed this. In contrast, Korean medicine doctors listened to my entire story rather than just depending on machines, which is why they were able to understand the problem. I also saw a significant improvement in my headaches. I used to suffer from severe headaches, and despite taking Western painkillers and Botox injections, there was very little improvement. However, my headaches significantly improved after acupuncture treatment, and now I do not need painkillers. I plan to continue receiving integrative treatment in future.”

#### 3.4.2. Case 3

“Receiving integrative treatment definitely makes a difference. I visit my Western medicine doctor only once every 2–3 months to get my prescriptions. Is this not too long for the patients? I am not even sure if the doctor will remember me, which makes me feel very uneasy. However, with Korean medical treatment between Western medicine appointments, I feel more at ease and find it easier to move around. I swim every morning and notice real changes since starting Korean medicine treatment. I can now perform swimming moves that I could not previously manage.”

#### 3.4.3. Case 4 (Caregiver's Statement)

“Since starting the integrative treatment, she has experienced falls less often. She used to constantly fall and bump into objects, and there was never a day without her body bruising. However, the number of falls significantly decreased after acupuncture. I have heard that dopamine medications often have many side effects, but my wife's dose has not increased over the years. I have seen that other people who take only dopamine medication have drastically increased their doses over time. I believe that integrative treatment may help slow the progression.”

#### 3.4.4. Case 7

“One day, I suddenly started seeing distorted windows and having hallucinations. I had just been to my Western medicine hospital, but my next appointment was in three months, and it was really hard to reschedule. Instead, I came to this clinic (Korean Medicine hospital) where I received acupuncture treatment. What I like about this place (Wonkwang University Korean Medicine Hospital) is that I can come anytime I feel uncomfortable. When I explained my symptoms, they immediately understood and asked if my medication dose had recently increased. When I saw my doctor at the previous hospital, I mentioned that my tremors and walking difficulties seemed to have worsened; therefore, my dopamine dosage increased. At the Korean Medicine clinic, we decided to reduce the dosage to the original level until my next Western medicine appointment, when the hallucinations disappeared. Later, when I saw a Western doctor, they agreed that this was the right thing. Of course, if I had returned to the hospital that prescribed the medication, they would probably have figured it out; however, rescheduling appointments would not have been easy. If it were not for this clinic, I might have struggled with the symptoms for months.”

## 4. Discussion

Most of the total long-term care costs incurred by patients with PD are attributed to hospitalization expenses, which is largely attributable to factors other than the neurological symptoms of the disease itself; instead, LEDD and polypharmacy are the primary contributors [6, 8]. The “honeymoon period” for PD lasts for approximately 5 years after onset. Thereafter, the efficacy of medications decreases, which leads to increased LEDD and the number of compensatory medications [6]. As the LEDD increases, so does the likelihood of adverse effects such as dyskinesia [7], and the probability of drug interactions exceeds 50% when taking five or more medications; this further increases the risk of side effects and potentially compromises dopaminergic medication efficacy [13]. Drug interactions are particularly relevant in PD management, as nondopaminergic medications that are used for symptom management can interfere with dopaminergic medication absorption, metabolism, or efficacy, necessitating LEDD increases to maintain therapeutic effects [13]. Therefore, controlling both LEDD and polypharmacy through comprehensive symptom management could potentially reduce hospitalization rates and thereby decrease the associated social costs.

This exploratory case series retrospectively analyzed the changes in LEDD and medication count in seven patients with PD who received integrative treatment for more than 5 years. Our findings indicate patterns that warrant further investigation. At baseline, patients had a mean LEDD of 386.43 ± 216.40 mg and medication count of 8.0 ± 4.93. At last follow-up, these decreased to 321.71 ± 219.21 mg and 6.14 ± 4.06, respectively, which represent mean reductions of 64.71 ± 62.97 mg in LEDD and 1.86 ± 1.35 in the medication count. The average LEDD for patients with PD who were receiving conventional treatment alone was 608.9 mg [10], and this included the LEDD of patients within 5 years of PD onset. Although direct comparisons were limited by differences in study populations and methodologies, all our participants with > 5 years postdiagnosis demonstrated lower final LEDD values than those typically reported in the literature [10]. The threshold for motor complications is generally considered to be an LEDD of 300 mg [6], and at follow-up, only two patients in our cohort exceeded this threshold, and neither exhibited motor complications. Regarding polypharmacy, baseline rates (85.7% for polypharmacy and 28.6% for hyperpolypharmacy) were higher than the previously reported averages of 40% and 18%, respectively [5]; however, these rates decreased substantially to 28.6% and 14.3% at follow-up. This can be considered a meaningful result, as previous studies [10] typically reported a mean H&Y stage of approximately 2.4 ± 0.8; however, our cohort—with more advanced disease (mean 2.71 ± 0.95)—showed lower final LEDD and reduced medication counts.

The potential mechanisms by which integrative treatment may contribute to LEDD stabilization and medication count reduction can be inferred from our observations and the existing literature. First, herbal medicine functions as a multitarget therapy owing to its composite ingredients, which can address multiple symptoms with a single formulation [14]. The effective management of nonmotor symptoms through Korean medicine interventions may reduce the need for additional symptomatic medications, directly addressing polypharmacy. In PD, the polypharmacy burden frequently stems not only from dopaminergic medications but also from medications prescribed for pain, gastrointestinal dysfunction, sleep disorders, and other comorbidities. For example, in Western medicine, dyspepsia and pain are typically treated with separate medications such as antacids and analgesics. However, a single herbal formulation alleviated dyspepsia and pain. In a Japanese case report, a patient with multiple symptoms, including dyspepsia, pain, and insomnia, managed to reduce 13 medications to just 2, including herbal medicine, and maintained this regimen for more than 10 years [15]. Second, gastrointestinal-targeted interventions may potentially optimize dopaminergic medication efficacy without requiring LEDD. Herbal medicines that act on the gastrointestinal system may stabilize the blood levels of dopaminergic agents [12], which potentially enhances the efficacy of these drugs without increasing the LEDD. In a study by Yakabi et al. [12], the herbal formula *Yukgunja-tang* (*Rikkunshito*, in Japanese, and *Liu Jun Zi Tang*, in Chinese) enhanced gastrointestinal motility by promoting gastric emptying and thereby stabilized blood levodopa levels. Similarly, in our case series, BIGT, which shares herbal constituents with *Yukgunja-tang*, was prescribed to three patients for gastrointestinal function optimization. In addition, GJDT was specifically administered to four patients to enhance their gastrointestinal motility and improve constipation. Interestingly, patients in our study who received these gastrointestinal-targeted herbal interventions along with EA demonstrated LEDD reduction, which suggests that improved gastrointestinal motility may enhance levodopa absorption and bioavailability. Acupuncture, particularly at specific acupoints, promotes gastrointestinal motility [16], which may help stabilize blood levels of dopaminergic agents. Recent evidence suggests that gut microbiota dysbiosis contributes to the PD pathophysiology through the microbiota–gut–brain axis and that modulation of gut flora with medicinal herbs can influence the neuroimmune and neuroendocrine pathways [17]. In this context, herbal prescriptions, such as BIGT and GJDT, may exert a therapeutic effect by improving gastrointestinal motility and potentially modulating the gut microbiota composition; these effects contribute to enhanced levodopa absorption and symptom control. Third, acupuncture and pharmacopuncture, which are used in Korean medicine, appear to improve various symptoms, such as pain and constipation, which potentially reduces the need for additional medication [11, 18, 19] and thereby prevents an increase in the medication count. Numerous reports have indicated that acupuncture is more effective than analgesics for managing pain [20, 21], which is a major complication of PD. In our case series, EA and ultrasound-guided acupuncture enabled discontinuation of multiple analgesic and gastrointestinal medications while maintaining symptom control [22, 23]. Acupuncture significantly improved UPDRS scores [11], and bee venom therapy may increase neurotrophic factor expression to potentially slow down the progression of PD [18]. This mechanism aligns with recent reviews that have highlighted oxidative stress and neuroinflammation pathways as therapeutic targets in PD [24]. The anti-inflammatory and antioxidant effects of bee venom pharmacopuncture may complement dopaminergic therapy and contribute to stabilizing both disease progression and medication requirements.

Finally, our approach inherently aligns with the principles of personalized and precise medicine. Recent developments in multimodal machine learning models and genomic profiling have emphasized the heterogeneity of PD and the need for individualized therapeutic strategies, including sex-sensitive considerations, for customizing optimal care [25]. Korean medicine naturally incorporates individualized symptom profiles, including gastrointestinal function, pain, and neuropsychiatric symptoms, into treatment decisions, and this aligns with precision medicine frameworks that integrate genetic variability, disease stage, comorbidity profiles, and sex-related factors [26].

Our findings are supported by emerging evidence that demonstrates the clinical relevance of integrative approaches in neurodegenerative diseases. Jin et al. [27] conducted a meta-analysis which showed that integrated Chinese and Western medicine significantly improved UPDRS scores and quality of life measures as compared to conventional Western medicine alone and reduced adverse reactions in patients with PD. This aligns with our clinical observations of improved symptom control and medication optimization. The integration of these approaches reflects the increasing emphasis on personalized and precision medicine for PD management. Korean medicine's constitution-based treatment approach naturally aligns with precision medicine principles, as both approaches emphasize individualized care based on patient-specific characteristics [26, 28]. In our cohort, integrative treatment was customized not only by core motor symptoms, but also based on individual nonmotor symptom profiles, including gastrointestinal dysfunction, pain, and neuropsychiatric symptoms. This personalized approach considers the heterogeneity of PD presentation and may contribute to a more effective management of both primary symptoms and medication-related complications, ultimately supporting the stabilization of dopaminergic medication requirements observed in our study.

This study had some important limitations that must be acknowledged. First, the small sample size limits the generalizability of our findings and precludes meaningful statistical analyses of treatment effects. Our participants may not be representative of the broader population of patients with PD, and the lack of a control group receiving conventional treatment alone prevents causal inferences regarding the effects of integrative treatment. However, this study focused on the outcomes of integrative treatment in terms of LEDD and polypharmacy, which have not been previously evaluated. Despite including only patients > 5 years postdiagnosis, when symptoms are typically more severe, we observed lower LEDD and polypharmacy rates than those reported in previous studies [10] in patients receiving conventional treatment alone. Second, this retrospective analysis could not establish a causation between integrative treatment and the observed changes in LEDD and medication counts. Heterogeneity in treatment approaches and individual patient factors makes it difficult to identify the specific interventions responsible for any observed changes. However, we observed that LEDD and polypharmacy rates at the last visit were significantly lower than those reported in the previous literature, and we presented specific cases and the existing literature [11, 12, 14–16, 18–22] to suggest that integrative treatment may contribute to the reduced LEDD and number of medications. Third, we did not systematically assess core PD symptoms or standardized outcome measures, such as UPDRS scores, which are essential for establishing clinical efficacy. However, we used the H&Y stage, which is a widely used and validated staging system for assessing disease severity and functional status in patients with PD [29].

Despite these limitations, this exploratory case series provides preliminary observations that may inform future research. The observed patterns suggest that comprehensive symptom management, which addresses both motor and nonmotor symptoms, may be important for optimizing the overall medication burden in patients with PD. Given that drug interactions have become increasingly probable with polypharmacy and can compromise the efficacy of dopaminergic medication, strategies that address multiple symptoms through fewer interventions may warrant further investigation. However, these findings should be interpreted as hypothesis-generating rather than as conclusive evidence. Future prospective studies with larger sample sizes, control groups, standardized outcome measures, and systematic assessments of both motor and nonmotor symptoms are necessary to evaluate the potential role of integrative treatment in long-term PD management and to establish any causal relationships between such interventions and medication optimization.

## Figures and Tables

**Table 1:** Clinicodemographic characteristics of the patients.

Variable		Number
Age, years	< 65	2
	≥ 65	5

Sex	Male	3
	Female	4

Disease duration, years	≥ 5 and < 10	3
	≥ 10	4

Start of CTWK	≥ 5 years from diagnosis	7
	< 5 years from diagnosis	0

H&Y stage	1	1
	2	1
	3	4
	4	1
	5	0

Comorbidities, by system	Cardiovascular	3
	Nervous	1
	Metabolic	4
	Psychiatric	2
	Urological	1

*Note:* CTWK, collaborative treatment with Western and Korean medicine.

Abbreviation: H&Y, Hoehn and Yahr.

**Table 2:** Individual patient data and treatment outcomes.

Case	Sex/age	DD (years)	DCTWK (years)	H&Y stage change	LEDD (mg) change	Total med count (KHM)^a^ change	Main symptoms	Details of KM treatment	Key outcomes
1	M/64	7	4	3 ⟶ 3	632 ⟶ 632	9 (0) ⟶ 6 (2)	Both hands tremor Bradykinesia Constipation Gait disorder Headache Jaw tremor Rigidity Sleep disorder	EA KHM (BIGT, GJDT)	Stable H&Y/LEDD maintained for 4 years. Eliminated gabapentin, tramadol, and acetaminophen via EA for headache control (NRS 8 ⟶ 3). Improved constipation by replacing magnesium hydroxide and lactulose with BIGT, GJDT.

2	M/74	12	8	2 ⟶ 2	200 ⟶ 200	17 (0) ⟶ 14 (0)	Bradykinesia Depressed mood Gait disorder Rigidity Nocturnal leg pain	EA BVP	Stable H&Y/LEDD was maintained for 8 years. Nocturnal leg pain was significantly alleviated (NRS 9 ⟶ 2) via EA and BVP, which enabled the discontinuation of pregabalin, tramadol, and piroxicam.

3	F/66	8.5	8	1 ⟶ 1	300 ⟶ 200 (discontinued rasagiline 1 mg)	3 (1) ⟶ 3 (1)	Constipation Left-hand tremor	EA BVP KHM (GJDT)	Stable H&Y/stage for 8 years. Achieved 100 mg LEDD reduction while maintaining medication count.

4	F/71	11	7	3 ⟶ 3	200 ⟶ 200	3 (0) ⟶ 3 (1)	Bradykinesia Dyspepsia Constipation Dysarthria Gait disorder Generalized tremor	EA BVP KHM (BIGT)	Stable H&Y stage and LEDD for 7 years. Improved dyspepsia by replacing rebamipide with BIGT.

5	F/67	9	5	3 ⟶ 3	750 ⟶ 650 (discontinued pramipexole 1 mg)	7 (0) ⟶ 4 (1)	Bradykinesia Constipation Gait disorder Left-hand tremor Perioral tremor	EA BVP KHM (GJDT)	Stable H&Y stage despite 100 mg LEDD reduction. Discontinued levosulpiride. Improved constipation by replacing bisacodyl/docusate sodium and magnesium hydroxide with GJDT.

6	M/64	10	9	3 ⟶ 3	300 ⟶ 150 (reduced levodopa 50 mg, pramipexole 0.25 mg, rasagiline 0.75 mg)	6 (0) ⟶ 4 (1)	Bradykinesia Constipation Gait disorder Left-hand tremor Postural instability Rigidity Urinary frequency	EA BVP KHM (GJDT) US-A (once)	Stable H&Y stage for 9 years. LEDD reduced from 300 to 150 mg through enhanced gastrointestinal function. Improved constipation via US-A and GJDT, which enabled the discontinuation of lactulose concentrate, magnesium hydroxide, and bisacodyl.

7	F/77	14	10	4 ⟶ 3	323 ⟶ 220 (discontinued pramipexole 0.25 mg, rasagiline 0.78 mg)	11 (0) ⟶ 9 (1)	Bradykinesia Constipation Depressed mood Dizziness Rigidity	EA KHM (BIGT)	103 mg LEDD reduction owing to hallucinations. H&Y stage and dizziness improved with BIGT therapy, which enabled the discontinuation of dimenhydrinate.

*Note:* Changes in the H&Y stage, LEDD, and medication count are expressed as “Baseline value ⟶ Last follow-up visit value.” Baseline was defined as the time of the patients' first visit for integrative treatment (Western–Korean collaborative care). BIGT, Bojungikgi-tang in Korean; Hochuekkito in Japanese; Buzhong Yi Qi Tang in Chinese; DCTWK, duration of collaborative treatment with Western and Korean medicine; years; EA. electroacupuncture; GJDT, Gyeji-ga-jakyak-daehwang-tang in Korean; Keishikashakuyakudaiotou in Japanese; Gui zhi jia shao yao da Huang Tang in Chinese.

Abbreviations: BVP, bee venom pharmacopuncture; DD, disease duration; H & Y, Hoehn and Yahr; KHM, Korean herbal medicine; KM, Korean medicine; LEDD, levodopa equivalent daily dose; US-A, ultrasound-guided acupuncture.

^a^Medication count includes Korean herbal medicines, which are indicated in parentheses. For example, 6 (2) indicates that the patient was taking six medications, of which 2 were Korean herbal medicines.

**Table 3:** Summary statistics of H&Y stage, LEDD, medication count, and duration of collaborative treatment with DCTWK.

Variable	Baseline visit	Last follow-up visit	Change (△)
	Median (IQR)/mean ± SD	Median (IQR)/mean ± SD	Mean ± SD
H&Y stage	3.0 (2.5–3.0)/2.71 ± 0.95	3.0 (2.5–3.0)/2.57 ± 0.79	−0.14 ± 0.38
LEDD (mg)	300.0 (250.0–477.5)/386.43 ± 216.40	200.0 (200.0–426.0)/321.71 ± 219.21	−64.71 ± 62.97
Medication count (no.)	7.0 (4.5–10)/8.0 ± 4.93	4.0 (3.5–7.5)/6.14 ± 4.06	−1.86 ± 1.35
DCTWK	—	8.0 (6.0–8.5)/7.29 ± 2.14	

*Note:* H&Y stage, LEDD, and medication count are presented at both the baseline (first visit for integrative treatment) and last follow-up visits, whereas DCTWK represents the treatment duration at the last follow-up visit only. Change (Δ) indicates the mean change (Last − Baseline) with standard deviation. To ensure comprehensive data representation, both median (IQR) and mean ± SD are provided, owing to the small sample size. IQR, interquartile range; DCTWK, duration of collaborative treatment with Western and Korean medicine.

Abbreviations: H&Y, Hoehn and Yahr; LEDD, levodopa equivalent daily dose; SD, standard deviation.

## Data Availability

The data supporting the findings of this study are available upon request from the corresponding authors. The data are not publicly available because of privacy and ethical restrictions.
